# High Intakes of [6S]-5-Methyltetrahydrofolic Acid Compared with Folic Acid during Pregnancy Programs Central and Peripheral Mechanisms Favouring Increased Food Intake and Body Weight of Mature Female Offspring

**DOI:** 10.3390/nu13051477

**Published:** 2021-04-27

**Authors:** Emanuela Pannia, Rola Hammoud, Ruslan Kubant, Jong Yup Sa, Rebecca Simonian, Brandi Wasek, Paula Ashcraft, Teodoro Bottiglieri, Zdenka Pausova, G. Harvey Anderson

**Affiliations:** 1 Department of Nutritional Sciences, Faculty of Medicine, University of Toronto, Toronto, ON M5S 1A8, Canada; e.pannia@mail.utoronto.ca (E.P.); rola.hammoud@mail.utoronto.ca (R.H.); ruslan.kubant@utoronto.ca (R.K.); terry.sa@mail.utoronto.ca (J.Y.S.); rebecca.simonian@mail.utoronto.ca (R.S.); zdenka.pausova@utoronto.ca (Z.P.); 2 Center of Metabolomics, Institute of Metabolic Disease, Baylor Scott and White Health, Dallas, TX 75226, USA; brandi.wasekpatterson@bswhealth.org (B.W.); paula.ashcraft@bswhealth.org (P.A.); teodoro.bottiglieri@bswhealth.org (T.B.); 3 Department of Physiology, Faculty of Medicine, University of Toronto, Toronto, ON M5S 1A8, Canada; 4 The Hospital for Sick Children, Toronto, ON M5G 0A4, Canada

**Keywords:** folic acid, [6S]-5-methyltetrahydrofolic acid, pregnancy, leptin, hypothalamus, metabolism, in utero programming, body weight, food intake, female offspring

## Abstract

Supplementation with [6S]-5-methyltetrahydrofolic acid (MTHF) is recommended as an alternative to folic acid (FA) in prenatal supplements. This study compared equimolar gestational FA and MTHF diets on energy regulation of female offspring. Wistar rats were fed an AIN-93G diet with recommended (2 mg/kg diet) or 5-fold (5X) intakes of MTHF or FA. At weaning, female offspring were fed a 45% fat diet until 19 weeks. The 5X-MTHF offspring had higher body weight (>15%), food intake (8%), light-cycle energy expenditure, and lower activity compared to 5X-FA offspring (*p* < 0.05). Both the 5X offspring had higher plasma levels of the anorectic hormone leptin at birth (60%) and at 19 weeks (40%), and lower liver weight and total liver lipids compared to the 1X offspring (*p* < 0.05). Hypothalamic mRNA expression of leptin receptor (*ObRb*) was lower, and of suppressor of cytokine signaling-3 (*Socs3*) was higher in the 5X-MTHF offspring (*p* < 0.05), suggesting central leptin dysregulation. In contrast, the 5X-FA offspring had higher expression of genes encoding for dopamine and GABA- neurotransmitter receptors (*p* < 0.01), consistent with their phenotype and reduced food intake. When fed folate diets at the requirement level, no differences were found due to form in the offspring. We conclude that MTHF compared to FA consumed at high levels in the gestational diets program central and peripheral mechanisms to favour increased weight gain in the offspring. These pre-clinical findings caution against high gestational intakes of folates of either form and encourage clinical trials examining their long-term health effects when consumed during pregnancy.

## 1. Introduction

The gestational period represents a critical window of growth, development, and plasticity during which environmental factors (e.g., diet) program the mother and offspring’s later-life health trajectory [1,2]. Folate is an essential micronutrient required during pregnancy for healthy brain development, function, and metabolic outcomes. While folic acid (FA) is the synthetic form incorporated into foods and prenatal supplements, it has no biological activity until it is reduced into the bioactive folate derivative, 5-methyltetrahydrofolate (5-MTHF). As an essential component of the 1-carbon cycle, 5-MTHF regulates nucleotide and monoaminergic neurotransmitter synthesis and signalling, regeneration of methionine from homocysteine, and provision of S-adenosylmethionine (SAM), which catalyzes methylation reactions [3]. However, the efficient metabolism of FA is dependent on genetic factors as well the amount consumed, with high intakes limiting its own metabolism and thus availability of 5-MTHF [4]. 

Pregnant women consume high amounts of FA in North America, with intakes reaching >2-fold above recommendations [5,6], and high unmetabolized FA have been found in both maternal plasma and umbilical cord samples [7]. Findings from clinical and pre-clinical studies support a potential contribution of high FA exposure to the pathogenesis of several diseases [8], including the development of obesity, diabetes, and the metabolic syndrome, possibly through epigenetic-dependent mechanisms. Adequate FA intake plays an important role in energy homeostasis as it is a determinant of neurodevelopment in the offspring [9] and of metabolic hormones such as leptin and insulin [10,11,12,13] that communicate energy status to the brain. Several rodent studies have also highlighted the dose-dependent relationship between FA and increased risk of developing characteristics of the metabolic syndrome. Male offspring exposed in utero to moderate or very high doses of FA, ranging between 2.5–20-fold requirements for rodents, exhibit higher food intake, body weight, visceral adiposity, and insulin resistance when fed either a control or obesogenic diet post-weaning. Associated with these responses is an increased maturation and expression of the orexigenic hypothalamic neuron expressing neuropeptide-Y (NPY) [14,15,16,17,18]. In contrast, female offspring may be more resistant to increased FA in the diet of the mothers and the metabolic consequence of obesity. Those exposed to moderately high FA (2.5–5-fold requirements) in utero show either no change or delayed body weight gain when fed a control diet post-weaning but have reduced bone health and an altered expression of hypothalamic energy regulatory genes [15,19,20]. However, when exposed to very high levels of gestational FA (20-fold), female offspring also exhibit higher body weight gain and food intake and are glucose intolerant [10].

The studies arising from human and animal models have challenged guidelines surrounding FA recommendations [21,22] and promoted usage of the naturally occurring calcium salt [6S]-5-methyltetrahydrofolic acid (MTHF) as an alternative. However, unlike FA, MTHF does not have a tolerable upper intake level nor an established conversion factor to express µg of MTHF to dietary folate equivalents (DFE). The FDA permits manufacturers to use their own conversion factor providing that it does not exceed that established for FA (1.7X) [23]. Thus, MTHF is often indicated on the Nutrition Facts Label to be at the equivalent dose of FA (1000 µg), which is 1.5-fold times the recommended 400 µg/day of DFE. Moreover, despite its marketed benefits as an alternative to FA, only recently has a comparison been made of its long-term effects during pregnancy [24]. In a comparison of equimolar MTHF vs. FA fed at 5X requirements to Wistar rats during pregnancy, post-partum mothers gained 75% more weight and ate 8% more food, as well exhibited evidence of leptin dysregulation [24]. RNA sequencing analysis of the arcuate nucleus of the hypothalamus (ARC) identified >250 genes that were differentially expressed at parturition between mothers fed the high MTHF compared to FA diet, many of which play a role in the development of obesity, food addiction, and metabolic dysregulation and are associated with their long-term phenotype. Candidate genes included those involved in hypothalamic dopaminergic, GABAergic, and glutamatergic signalling pathways that regulate not only the neurotransmission efficiency of energy regulatory neurons [25], but are important in modulating neurobehavioral and mood disorders during pregnancy. For the latter, treatment with high-dose MTHF supplementation (>5 mg/day) is recommended [26,27]. 

As pregnancy is a sensitive life-phase whereby even modest nutritional stressors may lead to adverse later-life consequences in the mother and/or child, it is important to continue to identify the modifiable risk factors. While most studies have mainly focused on male offspring, programming of the female offspring (i.e., mothers-to-be) towards metabolic dysfunction may contribute to the intergenerational cycle of several non-communicable diseases, as women who become pregnant with pre-existing metabolic conditions are at an increased risk of pathological pregnancies and programming of adult diseases [28]. Therefore, in this study, we aimed to determine the effect of folate form and dose during pregnancy on the female (F1) generation. We hypothesized that female offspring born to dams fed the MTHF form of folate at high levels would also be programmed with central and peripheral regulatory systems favouring higher food intake and body weight compared to those born to dams fed FA. Body weight gain to 19 weeks, food intake, energy expenditure, locomotor activity, plasma leptin, and glucoregulatory hormones and hypothalamic gene expression of leptin- and neurotransmitter signalling genes involved in energy regulation were measured. 

## 2. Materials and Methods

### 2.1. Animals and Diets 

First-time pregnant (2–3 days) Wistar rats weighing 170–250 g (Charles River Farms, QC, Canada) were singly housed in a 12 h dark-light cycle (lights on at 0700 hrs at 22 ± 1 °C) and provided ad libitum food and water throughout the study. The protocol (# 20011892) was approved by the University of Toronto Animal Care Committee. Upon arrival, dams (*n* = 16–18/group) were randomly allocated to one of four custom-made dietary interventions (Research Diets Inc., New Brunswick, NJ, USA) that were provided only during their 3-week pregnancy: an AIN-93G diet (energy density 4.0 kcal/g) containing FA at either recommended (1X-FA, 2 mg/kg diet, control) or high (5X-FA, 10 mg/kg diet) quantities for rodents or [6S]-5-methyltetrahydrofolic acid calcium salt (Metafolin^®^, Merck & Cie, Schaffhausen, Switzerland) at equimolar recommended (1X-MTHF, 2.1 mg/kg diet) or high quantities (5X-MTHF, 10.4 mg/kg diet). Diets were kept in the dark at 4 °C until freshly provided to the animals weekly. Diets were analytically tested for long-term stability [24]. FA and MTHF were provided at equimolar levels in the diet due to a suggested comparable bioavailability of MTHF at equimolar levels to FA. Equimolar levels are used in clinical trials comparing folate status and homocysteine level maintenance [29,30]. The FA form was used as a reference to calculate the MTHF equimolar dose equivalent as only the synthetic folate form contains a tolerable upper level (1000 µg/day). The recommended (1X) FA dose provides 2 mg/kg of FA (4000 kcal) and represents the basal requirement for rodents for adequate growth rate [31]. This dose reflects recommendations of 0.4 mg of FA/day for women at low risk of neural-tube defects planning a pregnancy [21]. The supplemented FA diet contains 5X the basal requirement for rodents or 10 mg/kg of FA (4000 kcal). This dose reflects FA intakes commonly consumed by pregnant women in North America during the post-fortification era at >2-fold above basal dietary requirements for humans [5,6]. As MTHF is incorporated at the equivalent dose as FA in supplements, intakes may soon increase beyond recommendations [22]. 

Upon parturition, a subset of dams and their entire litters (*n* = 5–6 dams/group) were terminated and one female pup per dam was used for analyses. Within 24 h of birth, litters from the remaining dams were culled to 6 pups (3 male/female) to reduce variability in the total milk available to pups. The control AIN-93G diet (1X-FA) was fed during lactation (3 weeks) until weaning (21 days post-birth) and then one female pup per dam (*n* = 10–12/group) was switched to a high-fat diet (45% kcal from lard) adjusted to contain a similar micronutrient content per 100 kcal as the control diet. The high-fat diet was used in this study to simulate the current dietary environment which is obesogenic for humans. All animals were monitored until 19 weeks post-weaning when they were terminated. 

### 2.2. Phenotypic and Biochemical Analyses

Body weight of pups were recorded at birth and body weight and food intake of female offspring were measured weekly thereafter from weaning until 19 weeks. Weekly weight gain was calculated as the difference from wean and cumulative food intake calculated as total food intake from wean to 19 weeks post-weaning. All animals were terminated following an 8–10-h daytime fast via decapitation. Total visceral (intra-abdominal) adipose tissue (VAT), which included retroperitoneal, perirenal, and periovarian fat pads and livers were dissected manually, weighed, and snap frozen. Tissue weight (g) was adjusted for final body weight (g) and expressed as a percentage (%). Whole brains were excised and frozen on dry ice. Trunk blood was collected, and plasma was separated and frozen immediately at −80 °C for future analyses. 

At birth and 19 weeks post-weaning, plasma was analyzed for fasting concentrations of glucose (Cat#10009582, Cayman Chemical Co., Ann Arbor, MI, USA), insulin (Cat#80-INSRT-E01, Alpco, Salem, NH, USA), leptin (Cat#EZRL-83K, EMD Millipore, Billerica, MA, USA), active ghrelin (Cat#EZRGRA-90K, EMD Millipore, Billerica, MA, USA), and plasma triglycerides (Cat#10010303, Cayman Chemical, Ann Arbor, MI, USA). Plasma leptin concentrations analyzed post-weaning were adjusted for VAT mass. As a surrogate marker of insulin resistance from basal (i.e., fasting) glucose and insulin, the homeostatic model assessment for insulin resistance (HOMA-IR) was calculated at birth and 19 weeks post-weaning as:

HOMA-IR = fasting glucose (in mg/dL) × fasting insulin (in μU/mL)]/2430

In addition, at 12 weeks post-weaning, a subset of offspring were challenged with an intraperitoneal bolus of insulin (0.75 IU of insulin/kg body weight; insulin tolerance test, ITT) following a 6-h daytime fast. Glucose concentrations were subsequently assayed at 0, 15, 30, 60, 90, and 120 min via tail prick (Accu-Chek^®^ Aviva glucometer, Roche Diagnostics, Laval, Canada). The rate constant for glucose disappearance (kITT) was calculated from 0–60 min using the formula:

kITT (%min/1) = (0.693/t_1/2_) × 100

Total liver lipids were quantified by the Folch method [32] and expressed as a % of total tissue weight (g). 

### 2.3. Folate and Related 1-Carbon Metabolites 

5-MTHF was measured in plasma of pups at birth and in plasma and liver at 19 weeks post-weaning [33,34]. Related 1-carbon metabolites including methionine, SAM, S-adenosylhomocysteine (SAH), cystathionine, choline, and betaine were also measured in plasma and liver at 19 weeks post-weaning by liquid chromatography tandem mass spectrometry [35] as well as plasma total homocysteine [36].

### 2.4. Energy Expenditure and Locomotor Activity 

The Comprehensive Lab Animal Monitoring System (CLAMS™, Columbus Instruments, Columbus, OH, USA) was used for the analysis of whole-body energy expenditure. At 14 weeks post-weaning, a subset of female offspring (*n* = 8/group) were transferred to The Hospital for Sick Children (Toronto, ON, Canada) and acclimatized to the new environment for 1 week before testing at 15 weeks post-weaning. Data were collected at room temperature and for two consecutive 24-h periods, but only data derived from the second 24-h period were used for analysis. Heat production was calculated automatically from O2 consumption (VO2) and CO2 production (VCO2) as follows: heat (kcal/kg/h) = (3.815 + 1.232 × (VCO2/VO2)) × VO2 (mL/kg/h) and animal activity including total (XTOT), ambulatory (XAMB), and stereotypy (ZTOT) activity measured as beam breaks/12 h (Opto-M3 activity monitor, Columbus Instruments, Columbus, OH, USA). 

### 2.5. Brain Dissections and qRT-PCR

To begin to determine the long-term programming effects of the gestational diets on central energy homeostatic pathways, hypothalamic genes that influence the neurotransmission of appetite regulating neurons were measured. The genes selected were also based on those found to be associated with increased post-weaning food intake and body weight in dams fed these diets during gestation [24]. Target genes were *Npy*, pro-opiomelanocortin *(Pomc)*, leptin receptor (*ObRb*), and downstream leptin signalling genes, including signal transducer and activator of transcription-3 (*Stat3*) and suppressor of cytokine signaling-3 (*Socs3*), as well dopamine receptor 2 (*Drd2*), gamma-aminobutyric acid type A receptors -4alpha *(Gabra4),* -delta *(Gabrd),* -epsilon *(Gabre),* and glutamate receptor-2 *(Grm2).*

Briefly, the ARC was macrodissected from frozen whole brain using the stereotaxic coordinates outlined in the Rat Brain in Stereotaxic Atlas (Bregma −2.12 mm to −2.56 mm) [37]. Tissue was homogenized on ice and RNA extraction was performed using Trizol reagent (Invitrogen, Grand Island, NY, USA). A total of 1000 ng of RNA quantified by NanoDropTM2000 Spectrophotometer (Thermo Scientific Inc., Wilmington, DE, USA) was used to synthesize cDNA using the HighCapacity cDNA Archive Kit (Applied Biosystems Inc; ABI, Foster City, CA, USA) on the TProfessional Standard Gradient 96 thermocycler (Biometra). qRT-PCR using SYBR Green (Thermo Fisher) was performed on the ABI PRISM 7900 Sequence Detection System. Beta-2-microglobulin (*B2m*) was used as a housekeeping gene. mRNA fold-changes were analyzed using the 2^-∆∆CT^ method and expressed as relative changes to the control (1X-FA) group. Primers to measure the expression of genes of interest were designed using Primer-BLAST software as shown in Table 1. 

### 2.6. Statistical Analyses

SAS Version 9.4 software (SAS Institute Inc., Carey, NC, USA) was used to analyze all data. A two-way analysis of variance (ANOVA) using PROC GLIMMIX procedure with gestational folate Dose (1X vs. 5X) and Form (FA vs. MTHF) as main factors and a Dose×Form interaction term was used to determine the gestational diet effect on weight gain, body weight, energy expenditure, metabolic markers, and hypothalamic regulatory genes. To identify whether body weight or food intake (dependent measures) were influenced by the transfer of animals between institutions, “batch transferred” was included as a covariate argument. To determine the relationship between final body weight and cumulative food intake, Pearson correlation with PROC CORR was used. To identify the effect of gestational diet on post-weaning weight gain over time, PROC GLIMMIX for repeated measures was used with gestational Diet and Time as the main factors and a Diet×Time interaction term. A Tukey’s post-hoc analysis adjusted for multiple comparisons followed all significant interactions. *p* < 0.05 was considered statistically significant. Data are expressed as least square (LS) Mean ± standard error of the mean (S.E.M.). 

## 3. Results

### 3.1. X-MTHF Offspring Gained more Weight and Ate more Food Than 5X-FA Offspring 

**Figure 1:**
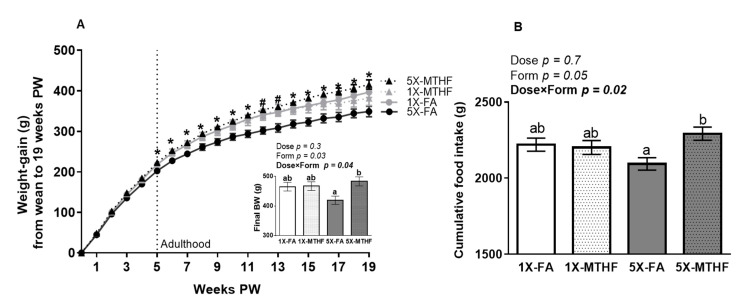
(**A**) Weekly body weight gain, final body weight (g), and (**B**) cumulative food intake (g) of female offspring fed a high-fat diet from wean to 19 weeks post-weaning (PW). Values are least square Mean ± standard error of the mean (S.E.M.), *n* = 10-12/group. Weight gain over time was calculated using a Repeated Measures ANOVA, Diet *p* = 0.02, Time *p* < 0.0001 and Diet×Time *p* < 0.001. Significant interaction effect was followed by Tukey post-hoc analysis. Significant differences (*p* < 0.05) between the 5X-FA and 5X-MTHF female offspring only are indicated by an asterisk (*). Significant differences (*p* < 0.05) between the 5X-FA compared to both the 5X-MTHF and 1X-MTHF offspring are indicated by a number symbol (#). Values in bold indicate significant effects. Final body weight and cumulative food intake analyzed by two-way ANOVA with Dose (1X or 5X) and Form (FA vs. MTHF) as main factors and a Dose×Form interaction term. Different letters (a,b) indicate significant differences at *p* < 0.05 by Tukey’s post-hoc analysis. Abbreviations: AIN-93G rodent diet with either 1X-FA, recommended folic acid (2 mg/kg diet); 5X-FA (10mg/kg diet); 1X-MTHF, equimolar recommended [6S]-5-methyltetrahydrofolic acid calcium salt (2.1 mg/kg diet); or 5X-MTHF (10.4 mg/kg diet). Dotted line represents post-weaning time point at which rodents become young adults.

Folate gestational diets did not adversely affect early-life growth, as the body weight of male (1X-FA 7.0 ± 0.2 g, 1X-MTHF 7.2 ± 0.1 g, 5X-FA 7.0 ± 0.2 g, 5X-MTHF 7.4 ± 0.2 g) and female (1X-FA 6.8 ± 0.1 g, 1X-MTHF 6.7 ± 0.2 g, 5X-FA 6.8 ± 0.1 g, 5X-MTHF 6.9 ± 0.2 g) pups at birth and the weight of female offspring from weaning throughout the peri-adolescent period were not different (Figure 1A). However, upon reaching maturity (5 weeks post-weaning), the 5X-MTHF female offspring gained consistently more weight than the 5X-FA offspring, but not compared to the 1X-FA or 1X-MTHF offspring (Diet *p* = 0.02, Time *p* < 0.0001 and Diet×Time *p* < 0.001, Figure 1A). A two-way ANOVA on final body weight confirmed that the effects of folate form were only apparent at the higher dose, as shown by a significant Dose×Form interaction effect (Dose *p* = 0.3, Form *p* = 0.03, Dose×Form *p* = 0.04). The 5X-MTHF offspring were 15% heavier than the 5X-FA offspring (*p* = 0.02), but not different from the 1X offspring. While the 1X folate offspring were also heavier than the 5X-FA offspring, these results did not reach statistical significance (~9%, *p* > 0.05).

A Dose×Form interaction effect was also found on measures of cumulative food intake up to 19 weeks post-weaning (Dose *p* = 0.7, Form *p* = 0.05, Dose×Form *p* = 0.02, Figure 1B). The 5X-MTHF female offspring consumed ~8% more food compared to the 5X-FA offspring, but food intakes of the 1X folate offspring were similar to both 5X groups. Cumulative food intake and total weight gain of the offspring were also strongly and positively correlated within each group (1X-FA r = 0.89, *p* < 0.001, 1X-MTHF r = 0.90, *p* < 0.001, 5X-FA r = 0.71, *p* < 0.01; 5X-MTHF r = 0.72, *p* < 0.01). 

**Figure 2:**
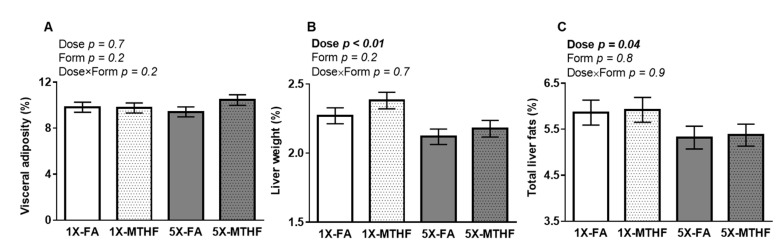
Percent (**A**) visceral adipose, (**B**) liver, and (**C**) total liver fat as adjusted per gram of final body weight in female offspring fed a high-fat diet for 19 weeks post-weaning. Values are least square Mean ± standard error of the mean (S.E.M.), *n* = 10-12/group. Analyzed by Two-way ANOVA with Dose (1X or 5X) and Form (FA vs. MTHF) as main factors and a Dose×Form interaction term. Significant at *p* < 0.05. Values in bold indicate significant effects. Abbreviations: AIN-93G rodent diet with either 1X-FA, recommended folic acid (2mg/kg diet); 5X-FA (10mg/kg diet); 1X-MTHF, equimolar recommended [6S]-5-methyltetrahydrofolic acid calcium salt (2.1mg/kg diet); or 5X-MTHF (10.4mg/kg diet).

Visceral adiposity was not different between the offspring as shown by a non-significant Dose, Form, and Dose×Form interaction effect (Figure 2A). In contrast, liver weight was affected by folate dose (Dose *p* < 0.01) but not form, with no interaction. Liver weight was ~8% lower in the 5X compared to 1X offspring of either folate form (Figure 2B). The lighter livers of the 5X offspring were reflected in their lower total liver fat (Figure 2C) compared to the 1X folate offspring (Dose *p* = 0.04, Form *p* = 0.8, Dose×Form *p* = 0.9)

### 3.2. X offspring have Higher Plasma Leptin at Birth and at 19 Weeks Post-Weaning Than 1X Offspring 

At birth and at 19 weeks post-weaning, plasma leptin concentrations were affected by folate dose (Dose *p* < 0.05), but not by form nor an interaction. Both forms of the 5X gestational diets led to offspring with higher plasma leptin at birth that was also higher until 19 weeks post-weaning (Table 1). As maternal circulating leptin contributes to fetal plasma leptin levels [38], a correlation analysis between plasma leptin at birth in the mother-offspring dyad was also performed using data collected from our recently published dataset [24]. While there was no correlation between the 1X maternal and offspring plasma leptins at birth, a strong and negative correlation was found between plasma leptin levels from the 5X folate groups, but this relationship was only significant in 5X-FA mother-offspring dyads (1X-FA *r* = −0.14, *p >* 0.05, 1X-MTHF *r* = 0.4, *p >* 0.05, 5X-FA *r* = −0.99, *p* < 0.01; 5X-MTHF *r* = −0.85, *p >* 0.05). 

A significant effect of folate dose (Dose *p* < 0.01) was also found on post-weaning plasma triglycerides, with 5X folate diets leading to lower plasma triglycerides compared to 1X diets, but these results were driven by the high FA form as indicated by a significant form effect (Form *p* = 0.02) but not an interaction effect. Plasma insulin, glucose, HOMA-IR, and active ghrelin at birth and 19 weeks post-weaning were not different between offspring groups (Table 2). kITT at 12 weeks post-weaning was also not different between groups (1X-FA 1.1 ± 0.16, 5X-FA 0.7 ± 0.15, 1X-MTHF 0.8 ± 0.16, 5X-MTHF 0.75 ± 0.15, *p* > 0.05).

### 3.3. Post-Weaning Energy Expenditure and Locomotor Activity Are Affected by Gestational Folate Diets

**Figure 3:**
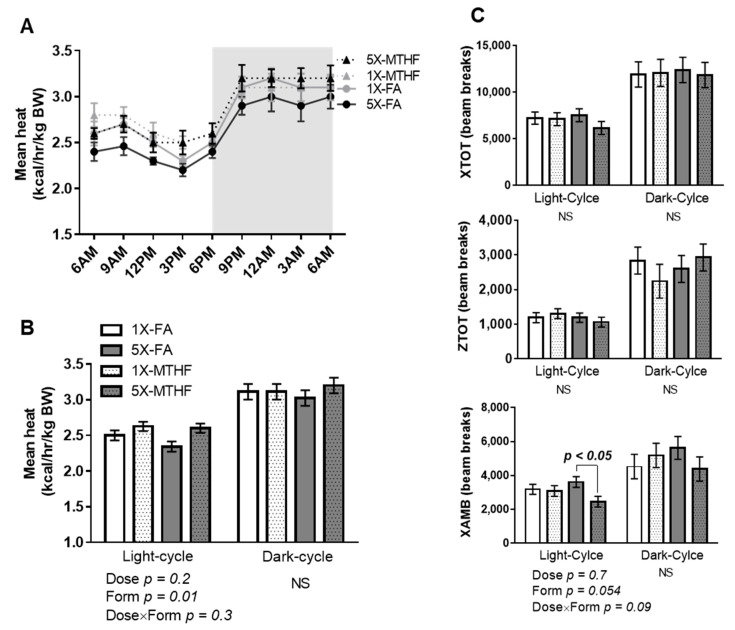
Mean (**A**) 24-h and (**B**) 12-h energy expenditure (kcal/h/kg body weight, BW) calculated as heat production of female offspring at 15 weeks post-weaning. (**C**) Total locomotor (XTOT), ambulatory (XAMB) and stereotypy (ZTOT) activity calculated as beam breaks every 12 h. Values are least square Mean ± standard error of the mean (S.E.M), *n* = 8/group. Twelve-hour energy expenditure and activity analyzed by two-way ANOVA with Dose (1X or 5X) and Form (FA vs. MTHF) as main factors and a Dose×Form interaction term. Significant at *p* < 0.05. Values in bold indicate significant effects. Abbreviations: AIN-93G rodent diet with either 1X-FA, recommended folic acid (2 mg/kg diet); 5X-FA (10 mg/kg diet); 1X-MTHF, equimolar recommended [6S]-5-methyltetrahydrofolic acid calcium salt (2.1 mg/kg diet); or 5X-MTHF (10.4 mg/kg diet); NS, non-significant; BW, body weight.

Mean energy expenditure calculated as heat production (kcal/hr/kg body weight) during the light-cycle was affected by folate form (Form *p* = 0.01) but not dose, nor their interaction. The MTHF offspring of either dose had higher energy expenditure during the light-cycle but not the dark-cycle compared to the FA offspring (Figure 3A,B). The form of folate also affected ambulatory activity as evidenced by a strong trend towards reduced activity that was mainly driven by the high MTHF form (Dose *p* = 0.7, Form *p* = 0.054, Dose×Form *p* = 0.09). Further analysis by student’s T-test stratified by dose confirmed that the 5X-FA offspring had higher ambulatory activity than the 5X-MTHF offspring (*p* = 0.006). Neither total activity (XTOT) nor stereotypy activity (ZTOT) was affected by the gestational diets (Figure 3C). Respiratory exchange ratio was also not different between the offspring, indicating the folate diets did not influence substrate oxidation (Light-cycle: 1X-FA 0.88 ± 0.01, 1X-MTHF 0.87 ± 0.01, 5X-FA 0.89 ± 0.01, 5X-MTHF 0.88 ± 0.01; Dark-cycle: 1X-FA 0.89 ± 0.01, 1X-MTHF 0.89 ± 0.01, 5X-FA 0.88 ± 0.01, 5X-MTHF 0.90 ± 0.01).

### 3.4. Post-Weaning Hypothalamic Energy Regulatory Genes Are Affected by Gestational Folate Diets

**Figure 4:**
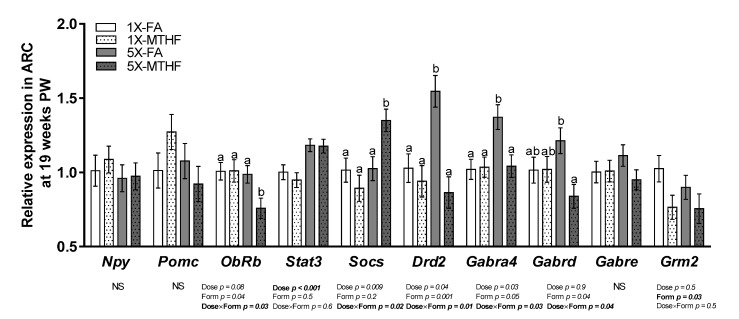
Relative mRNA expression of energy regulatory genes in the arcuate nucleus of the hypothalamus (ARC) of offspring at 19 weeks post-weaning (PW). *n* = 6–7/group. Values are least square Means ± standard error of the mean (S.E.M.). Analyzed by two-way ANOVA with Dose (1X or 5X) and Form (FA vs. MTHF) as main factors and a Dose×Form interaction term. Different letters (a,b) indicate significant differences at *p* < 0.05 by Tukey’s post-hoc analysis. Values in bold indicate significant effects. Abbreviations: AIN-93G rodent diet with either 1X-FA, recommended folic acid (2 mg/kg diet); 5X-FA (10 mg/kg diet); 1X-MTHF, equimolar recommended [6S]-5-methyltetrahydrofolic acid calcium salt (2.1 mg/kg diet); or 5X-MTHF (10.4 mg/kg diet). *Npy*, neuropeptide Y; *Pomc*, pro-opiomelacortin; *ObRb*, leptin receptor long isoform; *Stat3*, signal transducer and activator of transcription 3; *Socs3*, suppressor of cytokine signaling-3; *Drd2*, dopamine receptor type 2; *Gabra4*, gamma-aminobutyric acid type A receptor (*Gabr*) subunit 4alpha; *Gabrd*, *Gabr* subunit delta; *Gabre*, *Gabr* subunit epsilon; *Grm2*, glutamate receptor 2; NS, non-significant.

Hypothalamic energy regulatory genes were investigated in this study (Figure 4), many of which were also identified to be affected in the mother [24]. Of the 10 genes measured, seven were differentially affected by the gestational diets. *Stat3* was 1.2-fold higher in both 5X compared to the 1X offspring as evidenced by a significant dose effect (Dose *p* < 0.001) but not form, nor an interaction effect. Expression of *Grm2* was 0.8-fold higher in the FA compared to MTHF offspring (*p* = 0.03), but not by dose nor an interaction. However, *ObRb*, *Socs3*, *Drd2*, *Gabra4,* and *Gabrd* were affected by dose and form as shown by a Dose×Form interaction (*p* < 0.05). *ObRb* was 0.9-fold lower and *Socs3* was 1.4-fold higher in 5X-MTHF compared to all other groups (*p* < 0.05). In contrast, the 5X-FA offspring had 1.7- and 1.3-fold higher expression of *Drd2* and *Gabr4a,* respectively, compared to all other groups and 0.7-fold higher expression of *Gabrd* compared to the 5X-MTHF offspring only (*p* < 0.05). mRNA expression of *Npy*, *Pomc,* and *Gabre* were not significantly affected by treatments. 

### 3.5. Plasma 5-MTHF and Related 1-Carbon Metabolites Are Affected by Folate Gestational Diets

Plasma 5-MTHF concentrations in female pups at birth reflected the dose of folate in the gestational diets (*p* < 0.001), but there was no form nor an interaction effect (Table 3). Female pups born to mothers fed 5X folate diets had higher plasma 5-MTHF than those born to 1X diets. By 19 weeks post-weaning, plasma 5-MTHF was not different between diet groups nor were plasma methionine, SAM, SAH, or homocysteine. However, plasma cystathionine was higher in plasma of the 1X offspring than in the 5X offspring (*p* < 0.05). Neither form nor the interaction was a factor. Plasma betaine also tended to be affected by folate dose and form, with the 5X-MTHF offspring having lower concentrations than all other groups (Dose *p* = 0.06, Form *p* = 0.09, Dose×Form *p* = 0.3). Liver betaine concentrations were affected as reflected by the Dose×Form interaction (*p* < 0.04). Tukey’s post-hoc revealed the 5X-MTHF offspring had significantly lower liver betaine compared to the 5X-FA offspring, but similar to the 1X offspring. 5-MTHF and other related 1-carbon metabolites were not affected in the liver.

## 4. Discussion

**Figure 5:**
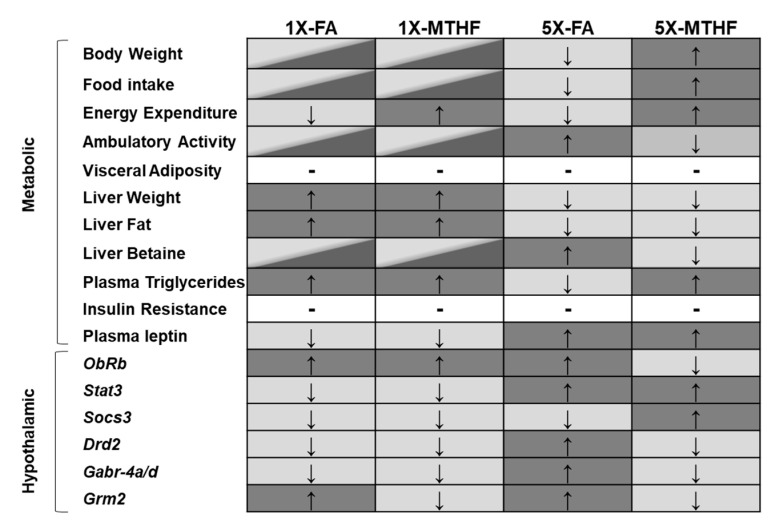
Summary of main metabolic and hypothalamic changes observed in offspring born to dams fed the gestational diets. Diet group panels that do not share the same colour reflect significant differences observed in the outcome measure. Panels that contain two colours have comparable outcome measures to both dark and light grey panels. Arrows indicate direction of change (↑increased, ↓decreased). Hyphen (-) indicates no difference between all groups Abbreviations: AIN-93G rodent diet with either 1X-FA, recommended folic acid (2 mg/kg diet); 5X-FA (10 mg/kg diet); 1X-MTHF, equimolar recommended [6S]-5-methyltetrahydrofolic acid calcium salt (2.1 mg/kg diet); or 5X-MTHF (10.4 mg/kg diet). ObRb, leptin receptor long isoform; Stat3, signal transducer and activator of transcription 3; Socs3, suppressor of cytokine signaling-3; Drd2, dopamine receptor type 2; Gabr-4a/d, gamma-aminobutyric acid type A receptors subunit 4alpha and delta; Grm2, glutamate receptor type-2.

This study confirms our hypothesis that female offspring born to dams fed the MTHF form of folate at high (5X) levels would be programmed with central and peripheral regulatory systems favouring higher food intake and body weight compared to those born to dams fed 5X-FA. The 5X-MTHF offspring had higher body weight gain, cumulative food intake, and light-cycle energy expenditure, as well as lower locomotor activity than the 5X-FA offspring. The metabolic hormones and hypothalamic regulatory genes assayed indicate plausible peripheral and central mechanisms by which the high folate diets may have affected in utero programming of body weight and food intake. A lasting effect of the maternal diet on altering peripheral signals is provided by measures of the anorectic hormone leptin that was higher in both 5X folate groups at birth and in later-life. However, differences in the programming effects by the gestational diets on pathways that are responsive to peripheral energy signals (i.e., leptin) were found. Of the 10 hypothalamic genes investigated, seven genes were differentially affected by the high maternal diets and are genes involved in food intake and metabolic control that associated with the offspring’s long-term phenotype. A summary of main findings is shown in Figure 5.

The observations that female offspring from mothers fed the 5X-MTHF compared to 5X-FA gestational diets had 15% higher body weight gain and 8% higher cumulative food intake are consistent with our previous reports. High (5X) gestational intakes of MTHF compared with FA increased post-weaning body weight and food intake in their mothers [24]. By comparison, a moderately high dose of gestational FA delays long-term body weight gain in mothers [24] and in female offspring [15,19], even when offspring are fed a normal fat post-weaning diet. As changes in body weights were not reflective of differences in visceral adiposity in neither the present study nor the others, future studies examining differences in the pattern of growth, body fat distribution and bone health are warranted. Moreover, the observation that the 1X folate diets resulted in female offspring with comparable body weights and food intake to both high folate groups to 19 weeks post-weaning is also in line with past reports in mothers [24]. A greater differentiation in body weight and food intake between the groups may have been achieved if folate was supplemented at higher levels (e.g., >10X). Nevertheless, the opposite later-life effects observed between FA and MTHF at moderately high doses support our conclusion that both folate dose and form consumed above recommended intakes are determinants of later-life health.

Final body weight and cumulative food intake of the offspring were strongly and positively correlated within each of the four diet groups, suggesting that higher weight gain of high MTHF compared to the FA offspring could be attributed to either increased food intake alone, or in addition to lower energy expenditure. However, lower energy expenditure at this time was not a factor. Offspring born to dams fed MTHF diets expended on average more energy during the light-cycle compared to those fed the FA diets. No differences were observed during the dark-cycle. However, this increase in energy expenditure was not sufficient to delay body weight gain in the 5X-MTHF group. Notably, higher light-cycle energy expenditure in rodents is an adaptive thermogenic response mediated by the sympathetic nervous system during the development of diet-induced obesity [39,40]. Thus, the changes in energy expenditure observed may indicate that MTHF additions to the gestational diet have a programming effect on adaptive thermogenesis, requiring future investigation. Additionally, a marginally significant form effect (*p* = 0.054) on ambulatory activity during the light-cycle was found that was largely driven by lower activity in 5X-MTHF compared to the 5X-FA offspring, but similar to 1X offspring; potentially contributing to their higher weight gain [41]. These results are also consistent with recent reports that show moderately high (5X) dose FA exposure throughout gestation to induce hyperactivity-like behavior in female [42] and male [43] offspring post-weaning. The body weight and food intake of the dams during pregnancy and lactation were not different between groups, nor were litter sizes or early-life growth of their offspring. Thus, the present study results support folate-induced in utero programming effects on the long-term phenotype of the offspring via central and peripheral energy regulatory mechanisms. 

A direct programming effect of the folate diets on fetal leptin expression and/or secretion is suggested and consistent with the known susceptibility of leptin to be epigenetically regulated due to environmental factors and nutritional status [44,45,46]. High gestational folate diets resulted in female offspring with 60% higher leptin at birth that was negatively correlated with that of their mothers. In the neonate, leptin is an important neurotrophic factor required for the development of food intake pathways, but does not exert anorectic actions until 4 weeks postnatal when downstream hypothalamic regulatory systems are developed [47]. During the early postnatal period, leptin may instead promote swallowing activity and hyperphagia to ensure adequate growth of the newborn. As well, the magnitude and timing of the surge of leptin is a determinant of the maturation of the immune system, lipid metabolism, endocrine stress, and reproductive axis response and bone formation [47,48,49]. Notably, our past studies report high gestational FA intakes to program lower femoral bone length, bone mineral content and density of female offspring associating with their lower long-term body weight [19]. Thus, higher leptin in 5X female offspring of either form may have resulted in several early-life physiological consequences contributing to differences observed upon maturity.

While no differences were observed in post-weaning measures of insulin resistance, female offspring born to dams fed high folate diets that had higher leptin at birth also exhibited 40% higher plasma leptin adjusted for VAT at 19 weeks post-weaning compared to 1X groups. As opposing observations were found on their body weight and food intake, these findings suggest different programming effects of the 5X gestational diets on pathways that are responsive to energy signals such as leptin that were sustained. This is confirmed by the observed differences between the offspring on central homeostatic regulatory pathways controlling food intake, body weight, and thermogenesis. Of the 10 target genes assayed in this study, seven were differentially expressed between offspring born to dams fed high MTHF compared to high FA gestational diets. Consistent with their higher body weight and food intake, the 5X-MTHF offspring exhibited differential expression in genes responsible for leptin signalling. In contrast, the 5X-FA diet affected the regulation of neurotransmitter signaling genes, as evidenced by up-regulation of genes encoding for dopamine and GABA- receptors that associated with their lower body weight and food intake. The differences in the molecular and metabolic programming effects of the two folate forms were only observed at the higher dose as the offspring exposed to recommended (1X) doses of either folate form exhibited comparable phenotypes.

In the ARC, leptin binds to the long-form leptin receptor (*ObRb*) which induces *Stat3* activation to mediate transcription of several genes within leptin-responsive neurons including the GABAergic NPY neurons which are appetite stimulating and the glutaminergic POMC neurons that are appetite supressing [25,50]. While subsequent transcription of *Socs3* is an important inhibitor of leptin signalling in this negative feedback pathway, chronic *Socs3* up-regulation is also sufficient to induce central leptin resistance [51]. Although expression of *Npy* and *Pomc* mRNA were not differentially affected in the ARC of offspring post-weaning, both 5X groups that had higher plasma leptin also had higher mRNA expression of the leptin signalling gene *Stat3* compared to the 1X offspring. In contrast, only the 5X-MTHF offspring had lower expression of *ObRb* and upregulation of *Socs3* mRNA, reflecting a possible disruption in ARC leptin signalling capacity that likely contributed to their long-term phenotype. However, this dysregulation may have been specific to the ARC (i.e., central leptin resistance) of the 5X-MTHF offspring. As evidenced by the compensatory increases in energy expenditure in the MTHF offspring, a role of leptin in mediating peripheral energy regulation is suggested, which may occur via the sympathetic nervous system during the development of obesity [5]. Moreover, lower total hepatic lipids in both 5X groups compared to the 1X offspring may be in part mediated by leptins actions in reducing total hepatic lipid content and de novo lipogenesis [52]. Together, these findings are in line with emerging evidence associating obesity with selective leptin resistance [53]. Noteworthy, as leptin is a pleiotropic hormone, our results do not exclude the possibility of other integrating signals contributing to the observed effects in the offspring and warrant investigation. 

This study further expands on our previous research showing that folate dose and form are determinants of the expression of genes encoding receptors in the GABAergic, glutamatergic, and dopaminergic pathways that regulate the neurotransmission efficiency of anorectic and orexigenic neurons. Several of the genes confirmed to be targets of folate-induced in utero programming effects on the mature female offspring in this study, were also targets of folate-induced effects on the maternal hypothalamus [24]. The 5X-FA offspring had 1.7-fold higher expression of *Drd2* and 1.3-fold higher expression of *Gabr4a* in the ARC compared to all other groups, as well exhibited up-regulation of *Gabrd* and *Grm2* mRNA compared to the 5X-MTHF offspring. Consistent with the phenotype of the 5X-FA offspring, higher expression of hypothalamic *Grm2* and *Drd2* mRNA associates with reduced feeding behaviour [54,55]. Moreover, consistent with retained ARC leptin signalling capacity and reduced food intake and energy expenditure in the 5X-FA offspring, the *Drd2* receptor isoform enhances the sensitivity of the anorectic response to leptin [56], is a key regulator of the negative feedback signal in prolactin secretion and determinant of food intake and body weight in females [57], and inhibits sympathetically-mediated brown adipose tissue thermogenesis [54]. In contrast, GABA, via actions on the multi-subunit GABA receptors, is associated with stimulatory effects on food intake [58]. While up-regulation of GABA_A_ receptor subunits may seem paradoxical in the 5X-FA offspring, the physiological functions of the receptor is dependent on its unique subunit composition [59] and their neuronal distribution [60]. While the 4α- and delta- subunits are co-localized throughout the brain, their specific roles in modulating food intake are not well described. However, it is known that the 4α- subunit exhibits high plasticity [61] and in the *anx/anx* mouse model of anorexia nervosa, there is a ~3-fold up-regulation in *Gabra4* in the hypothalamus [62]. Together, our findings confirm in utero programming effects of both 5X-FA and 5X-MTHF gestational diets leading to the dysregulation of central feeding pathways in female offspring post-weaning.

Since the 1-carbon cycle is responsive to the supplemented dose and form of folate and may contribute to the effects observed on body weight and food intake through many mechanisms, intermediates were measured in plasma and liver. At birth, both forms of the high folate dose led to higher plasma 5-MTHF in the offspring compared to recommended quantities, confirming the effectiveness of the gestational diets in transiently raising plasma folate levels as shown in the mothers. However, dose comparisons revealed that while high (5X) gestational FA increased offspring plasma 5-MTHF ~27% compared to 1X-FA diets, the high MTHF diet increased plasma 5-MTHF ~90% compared to the 1X-MTHF diet; consistent with reduced availability of 5-MTHF with high-dose FA compared to MTHF supplementation. While plasma folate concentrations were normalized between diet groups by 19 weeks post-weaning, the 5X folate offspring showed long-term reductions in plasma cystathionine, which may reflect potential alterations in the transsulfuration pathway. Also noted was a strong trend towards lower plasma betaine in the 5X folate offspring (*p* = 0.06) that was driven by the MTHF folate form, and significantly lower betaine in the liver of the 5X-MTHF compared to the 5X-FA offspring. These results are consistent with our recent data in showing 5X-MTHF compared to 5X-FA gestational diets result in lower plasma and brain betaine in mothers at birth that associate with dysregulation of hypothalamic energy regulatory signalling pathways [24]. As betaine is an alternative (i.e., folate-independent) methyl-donor in the 1-carbon pathway, high folate form-induced differences in betaine synthesis and utilization as a source of labile methyl groups in utero and throughout later development may have contributed to the differential programming effects of the 5X folate diets and warrants future investigation. 

The strength of this study arises from the identification of a role of folate form and dose in gestational diets on programming the phenotype of the female offspring and the association of their phenotype with altered expression of hypothalamic genes and response to a peripheral signal (i.e., leptin). However, a weakness arises from the limited number of mechanisms explored in the hypothalamus and periphery and thus follow-up analyses are needed. Moreover, comparative data on male vs. female offspring remain to be determined as well between offspring exposed to a high- vs. low-fat post-weaning diet. Nevertheless, our pre-clinical results utilizing physiological doses of FA and MTHF are relevant to informing decisions on the preferred form of folate to be used in supplements and in diets. It may be premature to prefer MTHF over FA without the support of clinical trials unless the amounts are more closely regulated to reflect requirements. 

## 5. Conclusions

MTHF compared to FA consumed at high levels in the gestational diet program central and peripheral mechanisms to favour increased weight gain in the female offspring. These pre-clinical findings caution against high gestational intakes of folates of either form, and encourage clinical trials examining their long-term health effects when consumed during pregnancy.

## Figures and Tables

**Table 1:** Primer sequences of target genes.

Gene	Forward Sequence (5’-3’)	Reverse Sequence (3’-5’)	Acc. No
*B2M*	GTGCTTGCCATTCAGAAAACTCC	AACTGAGACACGTAGCAGTTGAG	NM_012512.2
*Pomc*	GACCTCACCACGGAAAGCAA	TGTTCATCTCCGTTGCCTGG	NM_139326.3
*Npy*	TGGCCAGATACTACTCCGCT	TTCAAGCCTTGTTCTGGGGG	NM_012614.2
*ObRb*	CCAGTACCCAGAGCCAAAGT	GGATCGGGCTTCACAACAAGC	NM_012596.1
*Stat3*	TGCATTGATAAGGACTCTGGGG	CTGCCGTTGTTGGACTCCTC	NM_012747.2
*Socs3*	CCTCCAGCATCTTTGTCGGAAGAC	TACTGGTCCAGGAACTCCCGAATG	NM_053565.1
*Drd2*	GTTGTCTACCTGGAGGTGGTG	AGGTTCAGGATGCTTGCTGTG	NM_012547
*Gabra4*	ACAGTGATCCTTTCTCAAGTTTCC	CGTGAGGACTGTGGTTATTCCAA	NM_080587.3
*Gabrd*	TTTATACAGCATCCGCATCACCT	GAAGAGTAGCCATAGCTCTCCAG	NM_017289.2
*Gabre*	AGGAATTCTAAGAGGACCCAAGA	GCATCAATGGTCATCCTAACTGTG	NM_023091.1
*Grm2*	CTCCAGTGATTATCGGGTGCAG	TTCTGTGGCTGGAAAAGGATGAT	NM_001105711.1

Abbreviations: *B2M*, Beta-2-microglobulin; *Pomc*, pro-opiomelanocortin; *Npy*, Neuropeptide-Y; *ObRb*, leptin receptor long-form; *Stat3*, signal transducer and activator of transcription-3; *Socs3*, suppressor of cytokine signaling-3; *Drd2*, dopamine receptor-2; *Gabra4*, Gamma-aminobutyric acid type A receptor subunit-4alpha; *Gabrd*, Gamma-aminobutyric acid type A receptor subunit delta; *Gabre*, Gamma-aminobutyric acid type A receptor subunit epsilon; *Grm2,* Glutamate receptor-2.

**Table 2:** Plasma metabolic measures in female offspring at birth and 19 weeks post-weaning.

	1X						5X								
	1X-FA			1X-MTHF			5X-FA			5X-MTHF			Two-way ANOVA *p*-Value		
	Mean	±	S.E.M.	Mean	±	S.E.M.	Mean	±	S.E.M.	Mean	±	S.E.M.	Dose	Form	Dose × Form
Birth															
Leptin ng/mL	0.86	±	0.25	1.32	±	0.28	1.93	±	0.28	1.64	±	0.37	**0.03**	0.78	0.23
Insulin ng/mL	0.30	±	0.05	0.19	±	0.05	0.35	±	0.05	0.29	±	0.08	0.20	0.17	0.68
Glucose mg/dL	74.38	±	6.39	70.30	±	7.83	77.30	±	7.24	72.05	±	9.58	0.77	0.56	0.94
HOMA-IR	0.22	±	0.05	0.15	±	0.07	0.29	±	0.06	0.21	±	0.07	0.34	0.27	0.97
19 weeks PW															
Leptin ng/mL	14.08	±	2.12	11.15	±	2.12	17.18	±	1.95	18.35	±	2.12	**0.02**	0.67	0.33
Leptin/VAT	0.29	±	0.05	0.27	±	0.04	0.40	±	0.04	0.37	±	0.04	**0.02**	0.53	0.85
Ghrelin ng/mL	153.24	±	18.23	110.19	±	19.21	110.19	±	17.38	102.79	±	19.21	0.37	0.08	0.16
Insulin ng/mL	2.00	±	0.24	1.76	±	0.22	2.25	±	0.26	1.95	±	0.22	0.62	0.26	0.91
Glucose mg/dL	120.57	±	7.11	115.81	±	6.71	129.40	±	7.60	127.67	±	6.36	0.15	0.64	0.83
HOMA-IR	2.53	±	0.38	2.54	±	0.36	3.05	±	0.41	2.62	±	0.34	0.43	0.58	0.56
TG nmol/dL	70.01	±	7.88	83.08	±	7.37	45.17	±	7.88	70.08	±	7.88	**0.02**	**0.02**	0.45

Values are least square Mean ± standard error of the mean (S.E.M.), *n* = 8–10/group. Analyzed by two-way ANOVA with Dose (1X or 5X) and Form (FA vs. MTHF) as main factors and a Dose×Form interaction term. Significant at *p* < 0.05. Values in bold indicate significant effects. Abbreviations: AIN-93G rodent diet with either 1X-FA, recommended folic acid (2 mg/kg diet); 5X-FA (10 mg/kg diet); 1X-MTHF, equimolar recommended [6S]-5-methyltetrahydrofolic acid calcium salt (2.1 mg/kg diet); or 5X-MTHF (10.4 mg/kg diet); VAT, visceral adipose tissue; HOMA-IR, homeostatic model assessment insulin resistance calculated as follows: [fasting glucose (in mg/dL) fasting insulin (in U/mL)]/2430]; TG, triglycerides; PW, post-weaning.

**Table 3:** Plasma and liver 5-MTHF and related 1-carbon metabolite concentrations in female offspring at birth and 19 weeks post-weaning.

	1X						5X								
	1X-FA			1X-MTHF			5X-FA			5X-MTHF			Two-Way ANOVA *p*-Value		
	Mean	±	S.E.M.	Mean	±	S.E.M.	Mean	±	S.E.M.	Mean	±	S.E.M.	Dose	Form	Dose×Form
Birth Plasma															
5-MTHF (nmol/L)	217.0	±	25.34	174.80	±	25.34	275.60	±	25.34	331.2	±	25.34	**0.004**	0.81	0.09
19 weeks PW Plasma															
5-MTHF (nmol/L)	127.14	±	7.83	144.38	±	7.33	126.34	±	7.33	133.38	±	7.33	0.44	0.12	0.55
Met (µmol/L)	73.77	±	2.87	73.41	±	2.68	69.39	±	2.87	74.26	±	2.68	0.53	0.42	0.35
SAM (nmol/L)	269.02	±	11.77	269.87	±	11.77	267.97	±	12.58	248.94	±	11.77	0.37	0.45	0.41
SAH (nmol/L)	74.16	±	8.84	76.43	±	8.84	79.68	±	8.84	72.90	±	8.84	0.91	0.80	0.61
Homocysteine (µmol/L)	5.81	±	0.88	4.20	±	1.01	5.66	±	0.88	5.84	±	0.94	0.43	0.45	0.34
Cystathionine (nmol/L)	830.27	±	83.80	827.55	±	83.80	700.03	±	83.80	611.89	±	83.80	**0.04**	0.59	0.61
Choline (µmol/L)	10.89	±	0.72	11.36	±	0.72	10.70	±	0.72	10.34	±	0.72	0.41	0.94	0.57
Betaine (µmol/L)	90.71	±	7.53	86.57	±	6.25	85.18	±	6.68	67.06	±	6.25	0.06	0.09	0.29
19 weeks PW Liver															
5-MTHF (nmol/L)	27.78		1.63	32.31		1.63	29.57		1.63	30.75		1.63	0.94	0.09	0.31
Met (µmol/L)	447.80	±	45.17	447.88	±	45.17	506.25	±	45.17	508.25	±	45.17	0.20	0.98	0.98
SAM (nmol/L)	8.75	±	3.83	15.85	±	3.32	17.33	±	3.32	17.15	±	3.32	0.15	0.34	0.32
SAH (nmol/L)	15.55	±	3.36	21.78	±	2.91	18.41	±	2.91	21.56	±	2.91	0.67	0.13	0.61
Cystathionine (nmol/L)	25.48	±	2.51	23.77	±	2.51	26.61	±	2.51	26.99	±	2.51	0.51	0.95	0.54
Choline (µmol/L)	318.63	±	64.36	384.00	±	64.36	317.75	±	64.37	346.38	±	64.36	0.77	0.47	0.77
Betaine (µmol/L)	2458.75 ^ab^	±	233.63	2585 ^ab^	±	233.63	3045 ^a^	±	249.76	2230.75 ^b^	±	233.63	0.60	0.13	**0.04**

Values are least square Mean ± S.E.M., *n* = 6–7/group. Analyzed by two-way ANOVA with Dose (1X or 5X) and Form (FA vs. MTHF) as main factors and a Dose×Form interaction term. Significant at *p* < 0.05. Values in bold indicate significant effects. Abbreviations: AIN-93G rodent diet with either 1X-FA, recommended folic acid (2 mg/kg diet); 5X-FA (10mg/kg diet); 1X-MTHF, equimolar recommended [6S]-5-methyltetrahydrofolic acid calcium salt (2.1mg/kg diet); or 5X-MTHF (10.4 mg/kg diet); 5-MTHF, 5-methyltetrahydrofolate; SAM, S-adenosylmethionine; SAH, S-adenosylhomocysteine.

## Data Availability

All data presented in this study are contained within this article or extrapolated from our recently published manuscript Pannia et al. 2021. [6*S*]-5-Methyltetrahydrofolic Acid and Folic Acid Pregnancy Diets Differentially Program Metabolic Phenotype and Hypothalamic Gene Expression of Wistar Rat Dams Post-Birth. Nutrients. *13*(1), 48; https://doi.org/10.3390/nu13010048. Our previously published RNAseq dataset referred to in this study is openly available in NCBI Gene Expression Omnibus (GEO) at [https://www.ncbi.nlm.nih.gov/geo/query/acc.cgi?acc=GSE161954 (accessed on 25 December 2020)], reference number [GSE161954].
